# A Framework for Biomarkers of COVID-19 Based on Coordination of Speech-Production Subsystems

**DOI:** 10.1109/OJEMB.2020.2998051

**Published:** 2020-05-29

**Authors:** Thomas F. Quatieri, Tanya Talkar, Jeffrey S. Palmer

**Affiliations:** ^1^ MIT Lincoln Laboratory 57663 Lexington MA 02421 USA; ^2^ Harvard-MIT Speech and Hearing Bioscience and Technology ProgramHarvard Medical Sciences 438871 Boston MA 02115 USA; ^3^ MIT Lincoln Laboratory 57663 Lexington MA 02421 USA

**Keywords:** Asymptomatic, COVID-19, respiration, vocal subsystems, motor coordination

## Abstract

*Goal:* We propose a speech modeling and signal-processing framework to detect and track COVID-19 through asymptomatic and symptomatic stages. *Methods:* The approach is based on complexity of neuromotor coordination across speech subsystems involved in respiration, phonation and articulation, motivated by the distinct nature of COVID-19 involving lower (i.e., bronchial, diaphragm, lower tracheal) versus upper (i.e., laryngeal, pharyngeal, oral and nasal) respiratory tract inflammation, as well as by the growing evidence of the virus’ neurological manifestations. *Preliminary results:* An exploratory study with audio interviews of five subjects provides Cohen's d effect sizes between pre-COVID-19 (pre-exposure) and post-COVID-19 (after positive diagnosis but presumed asymptomatic) using: coordination of respiration (as measured through acoustic waveform amplitude) and laryngeal motion (fundamental frequency and cepstral peak prominence), and coordination of laryngeal and articulatory (formant center frequencies) motion. *Conclusions:* While there is a strong subject-dependence, the group-level morphology of effect sizes indicates a reduced complexity of subsystem coordination. Validation is needed with larger more controlled datasets and to address confounding influences such as different recording conditions, unbalanced data quantities, and changes in underlying vocal status from pre-to-post time recordings.

## I. Introduction

**Fig. 1.**
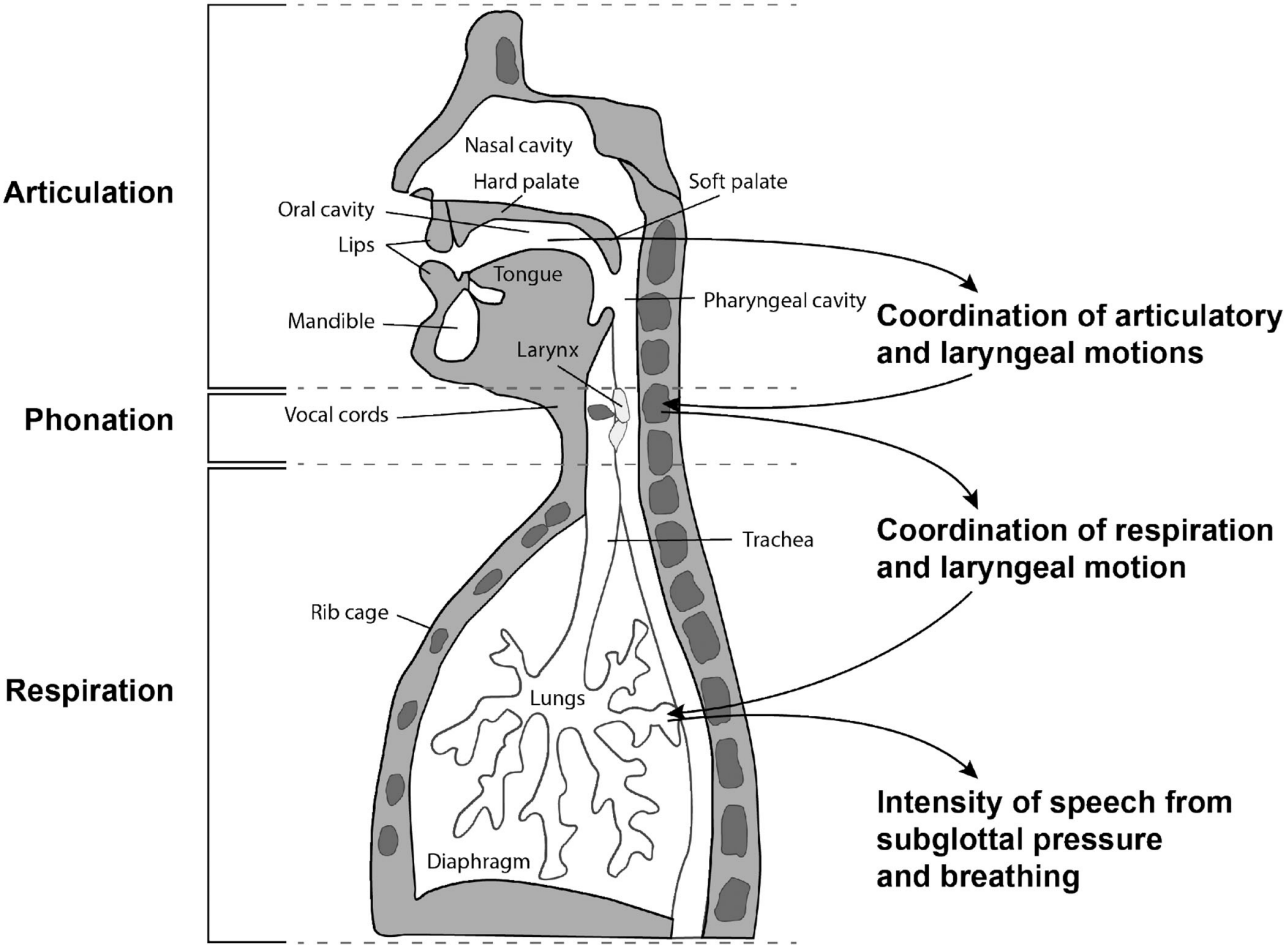
Speech subsystems and their coordination hypothesized to be affected by COVID-19.

Covid-19 is often characterized by specific dysfunction in respiratory physiology including the diaphragm and other parts of the lower respiratory tract, thereby affecting patterns of breathing during inhalation and exhalation of air from the lungs [1]. In speech production, during the exhalation stage, air from the lungs moves through the other essential vocal subsystems, i.e., through the trachea and larynx and into the vocal tract pharyngeal, oral and nasal cavities (Fig. 1). The manner in which we breathe in speaking, including the rate and length of an exhalation (coupled to the number of words in a phrase or sentence), and its intensity and variability, highly influences the quality of our voice. For example, the loudness, aspiration (‘breathiness’), steadiness of fundamental frequency or ‘pitch’ during phonation, and the mechanism by which we alter speaking rate all effect vocal quality. Furthermore, the respiratory system is highly coordinated with these primarily laryngeal-based subsystems [2], [3] . Likewise, in turn, laryngeal activity is finely coupled to articulation in the oral and nasal cavities [4]. Although impact on speech subsystems and their coordination are often perceptually obvious with a condition involving inflammation, these changes can be subtle in the asymptomatic stage of an illness, i.e., incubation and early prodromal periods, or in recovery.

In addition to the physiological manifestations of COVID-19, recently there have been reported symptoms that relate to temporary neuromuscular impairments [5], [6] and loss of taste and smell [7], [8] which can have implications for muscular control and proprioceptive feedback, respectively, in speech production. Given the physiologically-based insult to breathing functions, as well as this growing evidence of neurological deficits present in COVID-19, we hypothesize that biomarkers derived from measures of vocal subsystem coordination may provide a sensitive indicator of COVID-19, most importantly in its asymptomatic stage.

## II. Materials and Methods

### A. Dataset

Audio data for five subjects was obtained from YouTube, Instagram, and Twitter sources: pre-COVID-19 (before exposure) and post-COVID-19 (after positive diagnosis but presumed asymptomatic). Within the post-COVID-19 times of the interviews, chosen for analysis of subtle voice changes, there were no obvious symptoms (e.g, coughing or heavy breathing) by observation or self-report. Nevertheless, we could not know with certainty the full set of symptoms, nor if the subjects were presymptomatic. Subject-only regions were segmented manually from the videos to exclude secondary speakers such as interviewers or interviewees and other interferences. The recordings are taken from press conferences and TV interviews all with celebrities or broadcast hosts, typically using high-quality recording facilities. Though consistent environment and high signal quality were sought across pre- and post-states, the data can have varying environmental and recording conditions. Post-recording times were in the range of days with pre-recording times in the range of days-to-years. Signal-to-noise ratios were fairly high and consistent across pre- and post-conditions, ranging per-subject from about 18 to 10 dB. The Supplementary Material Section provides subject-specific and group statistics on segment durations and counts, pre- and post-recording times and environmental noise conditions.

### B. Subsystem Model

**Fig. 2.**
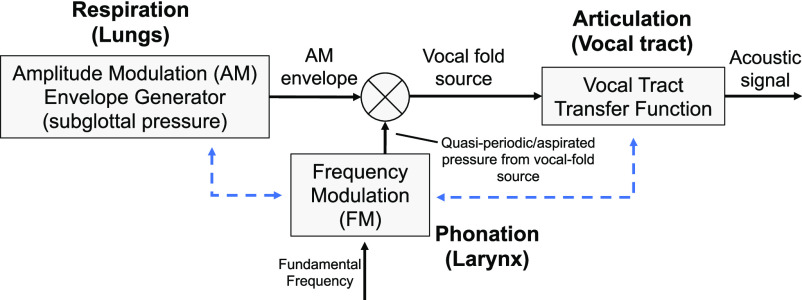
Fundamental speech-production subsystem model illustrating two of the potential points of coordination (dashed blue).

Our speech feature selection is based on the physiologically-motivated speech production model in Fig. 2 where the airflow from the lungs during the exhalation phase of speech production passes through the bronchial tubes through the trachea and into the larynx. The ‘intensity’ of the airflow (velocity), that we refer to in this note as the *respiratory intensity,* governs time-varying loudness, and is coupled (coordinated) with phonation, i.e., the vibration of the vocal folds (fundamental frequency or ‘pitch’), stability of phonation, and aspiration at the folds [3]. These characteristics are a function of laryngeal muscles and tissue, modulated by the respiratory intensity. Finally, in our model, the vocal fold source signal is modulated by, and coordinated with, the vocal tract movement during articulation.

### C. Feature Extraction

Standard *low-level* features associated with the various subsystems of Fig. 1 and Fig. 2 form the basis of our *high-level* features representing the coordination within and across these various subsystems and have been shown in previous research to be predictive of numerous neurocognitive conditions [9], [10], [11]*.*

Low-level univariate features characterize basic properties of the three vocal subsystem components. The speech envelope is used as a proxy for respiratory intensity and is estimated using an iterative time-domain signal envelope estimation algorithm, providing a smooth contour derived from amplitude peaks [12], [13]. At the laryngeal level, we estimate the fundamental frequency (pitch) using an autocorrelation approach [14], [15] and cepstral peak prominence (CPP), which provides stability of vocal fold vibration [16]. CPP is based on the ratio of the pitch-related cepstral peak relative to aspiration noise level and is a widely used and robust measure for assessing pathological speech [17], [18]. As a measure of vocal fold stability, CPP has the potential to reflect change in coupling of subglottal respiratory and laryngeal subsystems with COVID-19. Finally, formant center frequency tracks (vocal tract resonance frequencies), used as a proxy for articulation, relies on a robust Kalman-filter-based tracking algorithm [19]. Features are computed only during speaking using a speech activity detector [19].

High-level features involve multivariate auto- and cross-correlations of low-level features to produce measures of coordination within and across the underlying mechanisms of speech subsystems. Correlation functions for each (subject-only) segment are sampled at a time-delay scale of 10 ms. Within a segment, masking is applied to exclude speech pauses in computing correlations. The eigenspectra of each *correlation matrix*, formed from various sets of samples from correlation functions, quantifies and summarizes the relational properties of the set of feature trajectories.

Higher *complexity* of coordination across multiple channels is reflected in a more uniform distribution of eigenvalues, and more independent ‘modes’ of the underlying system components, while lower complexity is reflected in a larger proportion of the overall signal variability being concentrated in a small number of eigenvalues. In the latter case, the eigenspectra concentration typically manifests with high-rank eigenvalues being lower in amplitude and thus reflecting more dependent or ‘coupled’ system components. For the five subjects, independently and combined, Cohen's d effect sizes pre- versus post-COVID-19 were computed (for all segments in each category) based on eigenspectra using low-level respiration intensity, fundamental frequency, cepstral peak prominence, and formant center frequencies. More details about computing low- and high-level coordination features, effect sizes, and their interpretation are provided in the Supplementary Materials section.

## III. Results

**Fig. 3.**
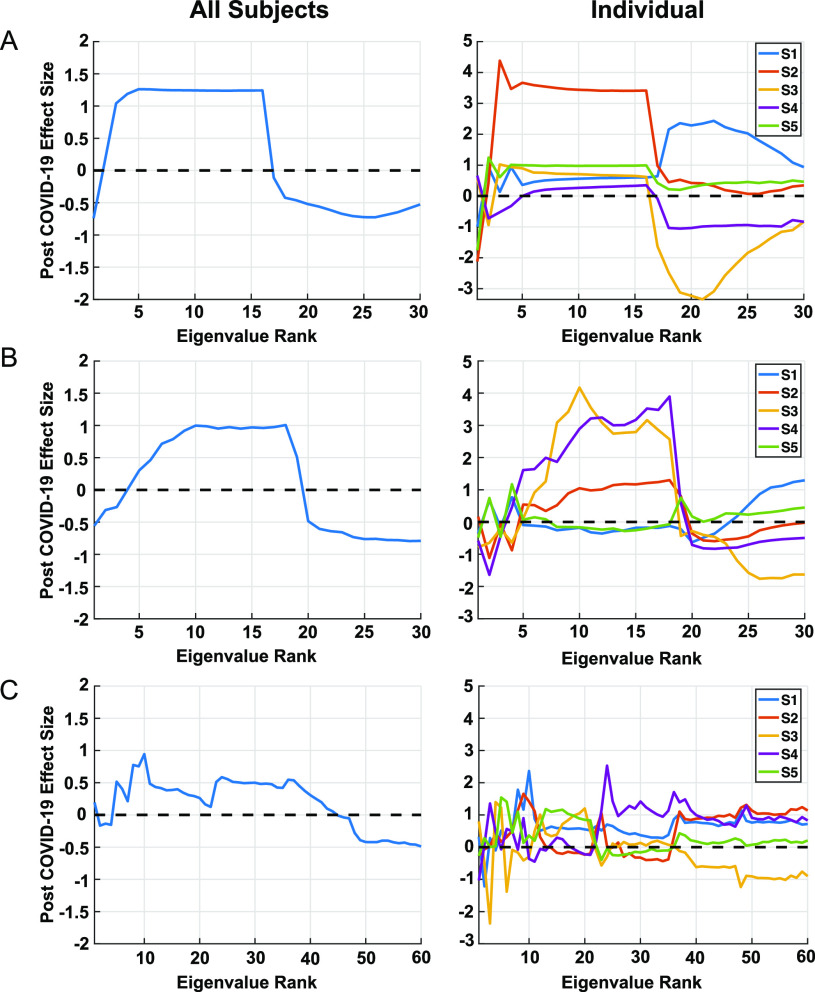
COVID-19 influence on coordination of respiration with laryngeal characteristics, as well as coordination of laryngeal characteristics with articulation. Group and subject-dependent effect sizes are shown in left and right columns, respectively. (A): respiratory intensity (speech envelope) and pitch (vocal fold fundamental frequency) − 30 eigenvalues obtained from 2 features × 15 correlation samples; (B): respiratory intensity and stability of vocal fold periodicity (CPP) − 30 eigenvalues obtained from 2 features × 15 correlation samples; (C): pitch and articulation (first 3 formant center frequencies) −60 eigenvalues obtained from 4 features × 15 correlation samples. Effect sizes greater in magnitude than 0.37 in the comparison across all subjects have corresponding p < 0.05.

The example given in Fig. 3, with pre-COVID-19 and post-COVID-19 (presumed asymptomatic) conditions, shows Cohen's d effect sizes with three measures of coordination: respiratory intensity (speech envelope) and pitch (vocal fold fundamental frequency), respiratory intensity and stability of vocal fold periodicity (CPP), and pitch and articulation (first 3 formant center frequencies). Effect size patterns for the two cases involving respiration show similar high-to-low trends across many of the subjects, with high-rank eigenvalues tending toward relatively lower energy for the post-COVID-19 cases. Effect sizes for the combined subjects indicate a similar but more distinct group-level morphology in these cases. On the other hand, effect sizes for coordination of pitch (fundamental frequency) and articulation (formant center frequencies) is more variable across subjects, but the combined counterpart shows a high-to-low trend, albeit weaker than those involving respiration. Although a strict interpretation is not possible due to the small cohort, at a group level, the morphology of effect sizes in Fig. 3 indicates a reduction in the complexity of coordinated subsystem movement, in the sense of less independence of coordinated respiratory and laryngeal motion and likewise, but to a lesser extent, for laryngeal and articulatory motion.

## IV. Discussion

Although the group-level eigenspectra-based effect size trends indicate a reduced complexity in coordination, clearly a larger cohort is warranted as well as addressing a number of confounders, including subject- and recording-dependences, in any validation procedure. For example, across all variables, inter-subject analysis shows for one subject a distinctly different trend of larger high-ranking eigenvalues that indicates a more complex but more erratic (or variable) coordination. Regarding signal quality, due to the nature of the online video sources, there is a variety of inter-and intra-subject recording variability, the most perceptually notable effect being reverberation, possibly modifying the true effect sizes, over- or under-estimating their importance. An example given in the Supplementary Material, isolating two of the subjects with more consistent, least reveberant environments, enhances the combined effect sizes relative to the N = 5 case.

## V. Conclusion

We have established a framework for discovery of vocal biomarkers of COVID-19 based on the coordination of subsystems of speech production involving respiration, phonation, and articulation. Our preliminary results, using a very limited data set, hint at support of the hypothesis that biomarkers derived from measures of vocal subsystem coordination provide an indicator, possibly in its asymptomatic stage, of COVID-19 impact on respiratory function. Given a sample size of five subjects and a growing list of COVID-19 symptoms, however, validation of our hypothesis will clearly require additional data and analysis to address potential confounders such as different recording environments and channels, unbalanced data quantities, and changes in underlying vocal status from pre-to-post time recordings. It will also be important to expand the suite of vocal features, introducing neurophysiological modeling of subsystem interactions, to address the increasing evidence of neurological insult arising from COVID-19 and feature specificity relative to typical flu and flu-like symptoms.

## VI. Supplementary Materials

The Supplementary Material section provides the following expansions of the main body topics: (1) more detailed description of the physiological motivation for the coordination model of Fig. 1 and Fig. 2; (2) more details of our standard low-level feature extraction, as well as introducing other vocal source features such as harmonic-to-noise ratio, vocal creak, and glottal open quotient; (3) effect sizes of summary statistics of the low-level features across the pre- and post-COVID-19 conditions as a comparative reference to the high-level feature effect sizes; (4) further description of the subject- and session-dependent environmental conditions; (5) more detailed description of the correlation methodology; and (6) expanded algorithm descriptions and software references to expedite use by others in the field.


