# Supplementary material for: A Framework for Biomarkers of COVID-19 Based on Coordination of Speech-Production Subsystems
Source: IEEE Open J Eng Med Biol. 2020 May 29;1:203–6. doi: 10.1109/OJEMB.2020.2998051 (PMC8975176; doi:10.1109/OJEMB.2020.2998051)
Supplement: Supplementary file 1 [file supp1-2998051.pdf]

Supplementary Materials

A Framework for Biomarkers of COVID-19 Based on Coordination of Speech-Production Subsystems

Thomas F. Quatieri, *Fellow*, Tanya Talkar, *Member*, and Jeffrey S. Palmer, *Senior Member IEEE* [1]

INTRODUCTION

The Supplementary Material section provides the following expansions of the main body topics: (1) more detailed description of the physiological motivation for the coordination model of Fig. 1 and Fig. 2; (2) more details of our standard low-level feature extraction, as well as introducing other vocal source features such as harmonic-to-noise ratio, vocal creak, and glottal open quotient; (3) effect sizes of summary statistics of the low-level features across the pre- and post-COVID-19 conditions as a comparative reference to the high-level feature effect sizes; (4) further description of the subject- and session-dependent environmental conditions; (5) more detailed description of the correlation methodology; and (6) expanded algorithm descriptions and software references to expedite use by others in the field.

I. MATERIALS AND METHODS

A. *Dataset Characterization*

As introduced in the main body, audio data for five subjects, pre-COVID-19 (before exposure) and post-COVID-19 (after positive but asymptomatic), was obtained from YouTube, Instagram, and Twitter sources. The recordings were taken from press conferences, TV shows, and TV interviews all with celebrities or broadcast hosts, typically using high-quality recording facilities, but in varying environmental conditions. Generally, recordings occur in multiple environments within a pre- or post-stage. For each recording session, the data was segmented manually from the videos excluding secondary speakers such as interviewers or interviewees and other interferences. Table 1 provides for each of the five subjects the number of pre- and post-diagnosis segments and average segment durations, and approximate pre- and post-diagnosis recording times. Although the range of pre-diagnosis times (days-to-years) is much greater than post-diagnosis times (days), because the subjects are all young- to mid-aged adults, voice change due to development or aging is not a concern. Nevertheless, there is always the possibility of a pathological state arising, e.g., nodules or polyps, over a long time stretch.

Table 1: Segment statistics of pre- and post-COVID-19 YouTube, Twitter, and Instagram videos.

Non è possibile visualizzare l'immagine collegata. Il file potrebbe essere stato spostato, rinominato o eliminato. Verificare che il collegamento punti al file e al percorso corretti.

Table 2: SNR statistics of mean and standard deviation (st.dev.) of segments across subjects pre- and post-COVID-19.

Non è possibile visualizzare l'immagine collegata. Il file potrebbe essere stato spostato, rinominato o eliminato. Verificare che il collegamento punti al file e al percorso corretti.

Table 2 provides a characterization of environmental conditions with respect to signal-to-noise ratio (SNR). Here we approximated SNR as the average signal energy during pauses (non-speech) divided by the average energy during speech in decibels (dB). Non-speech versus speech intervals were obtained using a standard speech activity detector [1]. On average there is a consistency across pre- and post-conditions per subject (about 1 dB difference) while the range of SNR from roughly 18 dB to 10 dB is on the order of noise levels we have encountered in using coordination measures effectively in tracking a variety of neurocognitive conditions [2][3]. (We note that all videos were mp3 encoded.) A stronger environmental effect is that of reverberation. Reverberation has proven more difficult to measure quantitatively, but the effect seems to stand out perceptually in some segments, for example, one involving a hall and a few others in home environments during quarantine. We return to the effect of reverberation in Section II.C.

[1] *DISTRIBUTION STATEMENT A. Approved for public release. Distribution is unlimited.*

*This material is based upon work supported by the Under Secretary of Defense for Research and Engineering under Air Force Contract No. FA8702-15-D-0001. Any opinions, findings, conclusions or recommendations expressed in this material are those of the author(s) and do not necessarily reflect the views of the Under Secretary of Defense for Research and Engineering.**© 2020 Massachusetts Institute of Technology. Delivered to the U.S. Government with Unlimited Rights, as defined in DFARS Part 252.227-7013 or 7014 (Feb 2014). Notwithstanding any copyright notice, U.S. Government rights in this work are defined by DFARS 252.227-7013 or DFARS 252.227-7014 as detailed above. Use of this work other than as specifically authorized by the U.S. Government may violate any copyrights that exist in this work.*

All three authors are affiliated with MIT Lincoln Laboratory. Thomas F. Quatieri and Tanya Talkar also have affiliation with the Harvard-MIT Speech and Hearing Bioscience and Technology Program, Harvard Medical Sciences.

It is also important to emphasize that due to the nature of the online video source (YouTube, Instagram, and Twitter), it is natural to consider the emotional context (e.g., the subjects' mood or stress level) that could confound the results. The subjects considered, however, routinely appear at press conferences and TV shows and interviews, so elevated heart rate and other confounders should be normalized, e.g., this isn't their first time in front of a camera, thus it is probably a consistent response for them. Nevertheless, there is the possibility of mood change such as due to self-knowledge of the COVID-19 diagnosis in the post- versus the pre-state. This is a consideration inherent in any longitudinal speech data collection.

B. Subsystem model

In the main Letter body, we presented a speech signal processing model that was physiologically motivated (Repeated here in Fig. 1.) In this model, coordination can occur both between the respiratory and laryngeal subsystems, and between the laryngeal and articulatory systems. It is hypothesized that this coordination across subsystems can change in the presence of COVID-19 either due to physiological insult to the respiratory system or to impaired neurological control of these subsystems. In this model, the 'intensity' of the airflow (velocity), that we referred to as *respiratory intensity*, governs time-varying loudness, and is coupled (coordinated) with phonation, i.e., the rate and nature of vibration of the vocal folds (pitch), stability of phonation, and aspiration at the folds [4]. The estimation of *low-level* features associated with each subsystem, and used as a basis for the *high-level* coordination features, is discussed in next section.

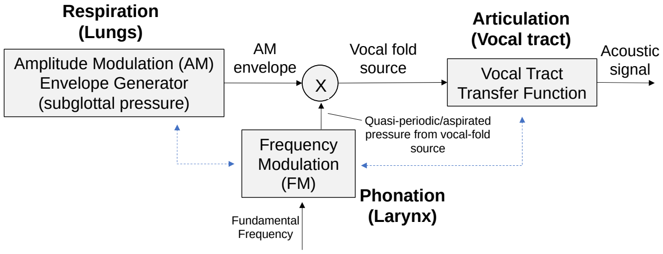

Fig. 1: Fundamental speech-production subsystem model and two of the potential points of coordination (dashed blue).

Stepping back momentarily for a deeper look into the model components, we see that respiratory intensity is modeled as an envelope that amplitude-modulates the airflow (induced by pressure in the lungs) that occurs at the vocal folds within the larynx. The airflow at the folds is either quasi-periodic or aspirated due to turbulence at the folds, amplitude-modulated by the respiratory envelope and is further modified by the vocal tract. Our assumption is that we can estimate the respiratory intensity as the envelope of the speech signal measured at the lip output..

C. Low-level feature extraction

We have found in previous work that the speech envelope is dominated by the respiratory intensity, but also contains a secondary resonance-harmonic interaction component that can occur as harmonics scan vocal tract resonances with time-varying pitch [5][6]. Nevertheless, the manner in which we extract the speech envelope yields a signal that approximately follows the speech signal's time-varying loudness [5][6] and is used as a proxy for respiration. Specifcially, the envelope was extracted using a custom MATLAB script that provides a smooth contour of amplitude peaks based on an iterative time-domain signal envelope estimation [5][6].

For the laryngeal subsystem, we compute fundamental frequency ('pitch') of the vocal fold vibration with an autocorrelation-based approach [7][8]. In addition, we compute many standard low-level features used in the speech community that characterize the nature of the vocal source. One such feature, cepstral peak prominence (CPP), captures the stability of periodicity in the vocal-fold vibration. The CPP is defined as the difference in dB between the magnitude of the highest peak and the noise floor in the power cepstrum which is the result of taking the inverse Fourier transform of the logarithm of the estimated spectrum of a signal [9]. Empirically, the CPP has been found to be robust in being used as an important measure of voice pathology and has been used effectively in neurocognitive applications [9][10][11]. Because the respiratory and laryngeal systems are coupled, the CPP can potentially capture changes in the degree of aperiodicity and aspiration at the vocal folds, that can change with insult to the respiratory system by COVID19.

Although possibly a less robust measure in the presence of a noise background, another more direct approach to getting at aspiration is through the harmonics-to-noise (HNR) ratio which is the ratio, in decibels (dB), of the power of the harmonic (periodic) signal from vocal-fold vibration and the power of the speech noise signal at the vocal folds created by turbulence as air rushes past the vocal folds from the lungs [12]. HNR is thought to reflect breathiness in a voice. Yet another vocal source measure is termed vocal creak, corresponding to what is often referred to as a 'creaky voice', which reflects large irregularity in pitch periods (typically with low average pitch) and high peakiness of airflow pulses that excite the vocal tract [13][14]. The value here is given as a creak probability. We have also have begun to explore the Glottal Open Quotient (GOQ) which is the ratio of the time duration over which the folds are open relative to the full glottal cycle. A larger GOQ often results in more turbulent airflow at the folds. Our fundamental frequency [8], CPP [15], and HNR [8] measures are obtained from the open source software package Praat [7] and the GOQ with the open source tool VOICEBOX [16].

Finally, our low-level features also aim to reflect the temporal dynamics of the articulatory component. As a proxy for articulation, a primary feature set is comprised of the vocal tract resonances, specifically the formant center frequencies, estimated by a Kalman filter technique, tracking the first three formant frequencies while also smoothly coasting through nonspeech regions. Here we use the open-source software package KARMA [1] to compute the first three formant frequencies (F1, F2, F3). Because of the embedded Kalman filtering, this method has proven robust in many applications

and compares well against other methods of formant estimation [17].

D. *High-level feature extraction*

Our high-level features, derived from low-level features, are designed to capture coordination of the temporal dynamics of speech production subsystems at different time scales for the two coordination points illustrated in the model of Fig. 1. This approach has been used for characterization of psychomotor slowing in depression, cognitive load and fatigue, and a variety of other neuromotor conditions [2][3].

Multivariate auto- and cross-correlations are computed within and across the underlying speech subsystems. We refer to this as *correlation structure*. Specifically, time-delay embedding is used to expand the dimensionality of the feature time series, resulting in a *correlation matrix* comprising auto- and cross-correlation patterns that represents coupling strengths across feature channels at multiple time delays. The eigenspectrum of the correlation matrix quantifies and summarizes the frequency properties of the set of feature trajectories. Higher complexity across multiple channels is reflected in a more uniform distribution of eigenvalues, with lower complexity reflected in a larger proportion of the overall signal variability being concentrated in a small number of eigenvalues, often with the high-rank eigenvalues being lower in amplitude.

For each speech segment, correlation matrices are calculated from various combinations of feature trajectories from the different speech subsystems. Each matrix contains the correlation coefficients between the time-series at specific time delays to create the embedding space. Each matrix is computed at a specific delay scale, i.e., time-sampling interval (e.g., 10, 30, 70, or 150 ms), with 15 time-delays used per scale. The delay scales allow for characterization of coupling of signals at different temporal resolutions. In the current work, eigenspectra were computed with a 10 ms delay scale and thus a relatively fine time resolution. Each matrix comparing n signals has a dimension of (n*15 x n*15). For all correlations, an automatic masking technique was used to include only speech within a segment, i.e., no pauses. The pipeline for the methodology is illustrated in Fig. 2 with the example of the generation of a formant frequency track correlation matrix based on the first 3 formants. Further mathematical details of this approach can be found in [2][3].

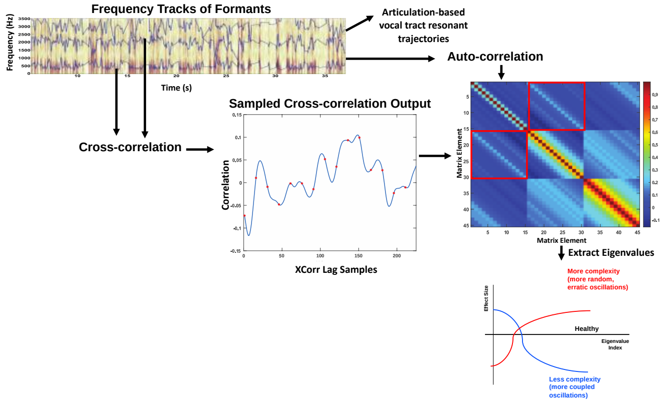

Fig. 2: Signal processing and feature extraction pipeline for eigenspectrum feature calculation. A caricature of effect sizes for three different states is shown: "healthy" or baseline (x-axis), high complexity (excessive mode independence or "erratic"), and low complexity (too little mode independence or "overly coupled").

E. *Effect size calculation*

In understanding the relative importance of a feature set, we compute the Cohen's d effect size as the difference of the pre- and post-condition feature means normalized by the average of the standard deviations:

$$Cohen's\ d = (M_{post} - M_{pre})/SD_{pooled} \qquad (1)$$

where:

M is the mean of each group

$$SD_{pooled} = \sqrt{(SD_{pre}^2 + SD_{post}^2)/2}$$

The right-bottom panel of Fig. 2 provides a caricature of eigenspectral effect sizes for three different states: 'healthy' or baseline (x-axis), high complexity (excessive mode independence or 'erratic'), and low complexity (too little mode independence or 'overly coupled').

For the five subjects, independently and combined, the Cohen's d effect sizes pre- versus post-COVID-19 were computed based on auto- and cross-correlation for a variety of vocal features. These measures are obtained from low-level respiration intensity, fundamental frequency, cepstral peak prominence, and formant center frequencies. Eigenvalues were computed for each audio segment. The effect sizes are then compared between the pre-segments and the post-segments, and the total number of segments depends on the comparison being made. The group level, i.e., combined subjects, averages out differences within an individual and reveals trends at a higher level that is likely more robust than for any one subject. There are obviously individual differences depending on recording and also day-to-day variance, but the group comparison aims to get at a higher-level pattern.

II. Results

In this section, effect sizes are computed first for low-level (univariate) features as a comparative reference for the high-

level coordination-based (multivariate) features. Low-level features include fundamental frequency, CPP, HNR, GOQ, and formant center frequencies. We then proceed to expand on coordination results of the main Letter, investigating (1) effect sizes associated with the (auto)correlation structure of respiratory intensity (via the speech envelope) and (2) a glimpse into environmental effects by looking at signal-to-noise ratio (SNR) for a subset of subjects.

A. *Univariate feature summary statistics*

Tables 3 and 4 provide mean and standard deviation summary statistics of the conventional univariate low-level features associated with phonatory and articulatory vocal subsystems. Statistics are shown for differences across the pre- and post-COVID-19 state and are denoted by ** for p values less than 0.05.

Table 3: Mean and standard deviation statistics for univariate vocal features. Statistics are shown for differences across the pre- and post-COVID-19.

	Laryngeal					Articulatory	
	F0	HNR	CPP	Creak	GOQ	F1	F2
Pre-COVID-19 (mean/st. dev.)	129.81/ 29.86	-15.73/ 67.42	19.19/ 3.63	0.05/ 0.10	0.701/ 0.01	462.06/ 176.01	1746.02/ 369.29
Post-COVID-19 (mean/st. dev.)	116.28/ 21.48	-29.33/ 77.75	18.14/ 3.11	0.08/ 0.12	0.701/ 0.01	417.36/ 221.13	1814.56/ 392.72

Table 4: Cohen's d effect sizes for univariate vocal features for statistics of Table 3. Effect size values are shown for differences across the pre- and post-COVID-19 state. Features with p < 0.05 are marked with **.

Effect Size	Laryngeal					Articulatory	
Post vs Pre	F0	HNR	CPP	Creak	GOQ	F1	F2
Mean	-0.50**	-0.85**	-0.63**	0.48**	-0.22	-0.51**	0.44**
St. Dev.	-0.75**	0.64**	-0.81**	0.24	-0.30	0.73**	0.29

For the laryngeal-based variables, we see a lowering of average pitch perhaps consistent with heavier, less pliant vocal folds due to inflammation, while the smaller CPP and larger creak probability with the post-condition may be associated with a more erratic vocal-fold vibration. A lower HNR may be associated with increased aspiration in the speech signal due perhaps to atypical closing of the folds. However, the glottal open quotient (GOQ) (notoriously most difficult to measure especially with background noise), is not consistent with an atypical closing of the folds and barely changes, not showing significance (p>0.05). The changes in F1 and F2 mean and standard deviation have no intuitive interpretation.

B. *Correlation structure of respiration*

As with correlation structure across vocal subsystems, we can explore the (auto)correlation structure within a single component trajectory of a subsystem. Given the importance of respiration in the COVID-19 context, Fig. 3 provides the eigenspectral-based effect size associated with respiratory intensity for the group and individual subjects with high-rank eigenvalues tending lower for the post-COVID-19 cases, indicating perhaps less independence of movement.

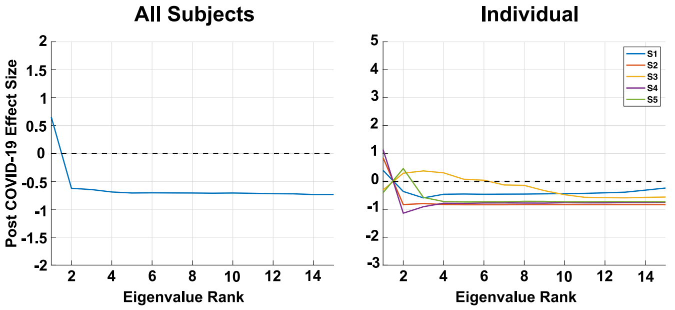

Fig. 3: Influence of COVID-19 on coordination of respiratory intensity (as measured through the speech envelope). Effect sizes greater in magnitude than 0.37 in the comparison across all subjects have corresponding p < 0.05.

C. *Subject-dependent correlation structure*

Regarding signal quality, due to the nature of the online video sources,we have noted there is a variety of inter- and intra-subject recording variability. Given that background noise levels are typically low in the given cases, the most perceptually notable effect is reverberation, possibly modifying the true effect sizes, over- or underestimating their importance.

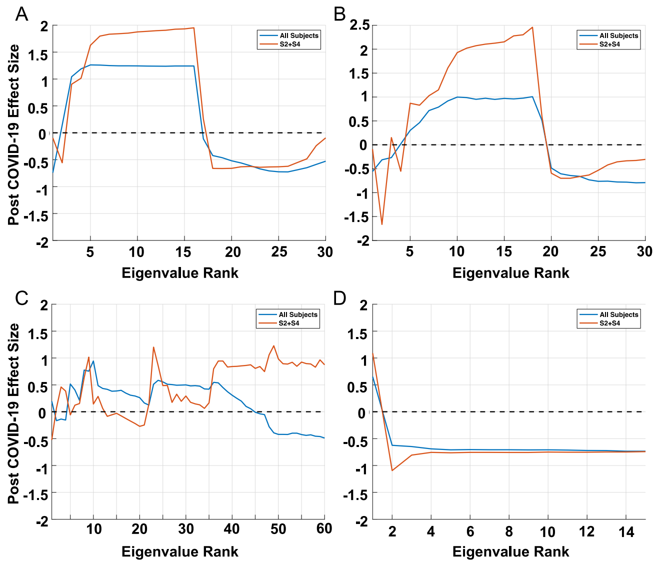

Fig. 4: Effect size comparison for eigenspectra of full group (N=5) versus two subjects combined with perceptually similar reverberant conditions. Clockwise from upper left: (A) respiratory intensity (as measured through the speech envelope) and pitch (fundamental frequency) (#eigenvalues = 30 = 2 features x 15 delays); (B) respiratory intensity and CPP (#eigenvalues = 30 = 2 features x 15 delays); (C) pitch (fundamental frequency) and articulation (3 formant center frequencies) (#eigenvalues = 60 = 4 features x 15 delays) ; (D) respiration intensity (#eigenvalues = 15 = 1 feature x 15 delays). Effect sizes greater in magnitude than 0.37 have corresponding p < 0.05.

An example isolating two of the subjects with more consistent, least reverberant environments (as determined perceptually), shown in Fig. 4, enhances the combined effect sizes relative to

the N=5 case. Specifically, for the combined two selected subjects, coordination measures involving respiratory intensity show a stronger reduction in complexity of coordination, while coordination of fundamental frequency with articulation shows an increased variability relative to the full N=5 case.

III. Discussion and Conclusions

This exploratory study has provided a framework to investigate coordination-based vocal biomarkers for early warning and tracking of COVID-19. Our univariate summary statistics show generally small but interpretable effect sizes for mean and/or standard deviation that may be consistent with the pre- versus post-COVID-19 conditions. Such features could be used in conjunction with coordination-based features.

Our effect sizes associated with coordination of respiratory intensity (as measured through the speech envelope) with laryngeal activitiy indicate lower complexity and thus a breathing pattern and laryngeal coordination with less variability than typical during speaking. On the other hand, coordination of laryngeal and articulatory activity revealed the opposite effect with apparently larger variability. Although interpretations of some of these differences may be consistent with the effect of inflammation in COVID-19 and reported neurological motor deficits, it is too early to draw conclusions given the limited sample size and lack of physiological ground truth. It is interesting to note, however, that at a group level reduced complexity in coordination based on similar correlation-structure features has been observed within various vocal subsystems for neuromotor conditions of Parkinson's disease, ALS, dementia, and TBI [2] while greater variability of complexity has been observed in conditions of cognitive fatigue [18] and hypoxia [19].

At a fundmental level, there needs to be a physiological interpretation and meaning attached to our features. It's important to stress that we are using formant center frequencies as a proxy for articulation and loudness as a proxy for respiratory intensity and so it will be important in future work to connect with these motor functions more explicitly. For example, we are currrently using a method of waveform-to-articulatory inversion developed in the context of Major Depressive Disorder (MDD) to measure coordination of articulatory trajectiories (key points of movement of the tongue, lips, and jaw). Here, we are finding similar but enhanced effect size morphologies relative to formant coordination [20]. Note that GOQ, albeit a sensitive measure, is also a means to get at the physiological level, e.g., glottal airflow patterns, through inverse filtering methods. Nevertheless, ideally, we seek independent physiological measures of, for example, breathing patterns, lung capacity, and nasal obstruction that provide ground truth against which the analysis data can be compared.

Regarding signal quality, due to the nature of the online video sources, in addition to the subject's mood and stress levels alluded to earlier, other effects such as the acoustic environment and other forms of room changes, can clearly influence results, over- or underestimating the effect size importance. For example, given the quarantine requirement after testing positive and multiple pre- and post-interviews per subject, there is a variety of inter- and intra-subject recording variability, including mood change due to self-knowledge of the COVID-19 diagnosis. Because the recordings were typically made with high-quality microphones, noise levels in the recordings were fairly well controlled. As noted, the more noticeable effect, however, is occasional reverberation. As we discussed earlier, isolating two of the subjects with more consistent, least reverberant environments enhances the combined effect sizes relative to the N=5 case.

This short note has clearly raised more questions than answers but, in facing the above challenges, the authors hope the proposed framework and exploratory results motivate others to investigate these questions with larger and more controlled datasets toward vocal biomarkers for early warning and tracking of COVID-19. An approach to biomarkers based on speech lends itself to nonintrusive widespread use through mobile devices and thus longitudinal tracking in naturalistic environments through pre- and post-diagnosis of COVID-19. If in addition to being sensitive in asymptomatic stages of the virus, the vocal biomarkers are also proven specific, especially with other nonintrusive sensing, the approach would help provide a key capability for early warning to interrupt and treat the disease before its rapid spread.

References

[1] D. D. Mehta, D. Rudoy, and P. J. Wolfe, "Kalman-based autoregressive moving average modeling and inference for formant and antiformant tracking," *J. Acoust. Soc. Am.*, vol. 132, no. 3, pp. 1732–1746, Sep. 2012, doi: 10.1121/1.4739462.

[2] T. F. Quatieri , J. Williamson, C. Smalt, J. Perricone, T. Patel, L. Brattain, B. Helfer, D. Mehta, J. Palmer, K. Heaton, and M. E. Moran., "Multimodal Biomarkers to Discriminate Cognitive State," in *The Role of Technology in Clinical Neuropsychology*, R. L. Kane and T. D. Parsons, Eds. Oxford University Press, 2017, p. 409.

[3] J. R. Williamson, D. Young, A. A. Nierenberg, J. Niemi, B. S. Helfer, and T. F. Quatieri, "Tracking depression severity from audio and video based on speech articulatory coordination," *Comput. Speech Lang.*, vol. 55, pp. 40–56, 2019, doi: 10.1016/j.csl.2018.08.004.

[4] Z. Zhang, "Respiratory Laryngeal Coordination in Airflow Conservation and Reduction of Respiratory Effort of Phonation," *J. Voice*, vol. 30, no. 6, pp. 760.e7-760.e13, Nov. 2016, doi: 10.1016/j.jvoice.2015.09.015.

[5] A. Röbel and X. Rodet, "Efficient spectral envelope estimation and its application to pitch shifting and envelope preservation," in *8th International Conference on Digital Audio Effects, DAFx 2005 - Proceedings*, 2005, pp. 30–35, Accessed: May 02, 2020. [Online]. Available: https://hal.archives-ouvertes.fr/hal-01161334.

[6] R. L. Horwitz-Martin, T. F. Quatieri, E. Godoy, and J. R. Williamson, "A vocal modulation model with application to predicting depression severity," in *BSN 2016 - 13th Annual Body Sensor Networks Conference*, 2016, pp. 247–253, doi: 10.1109/BSN.2016.7516268.

[7] P. Boersma and D. Weenink, "Praat: Doing phonetics by computer [Computer program]," 2018. http://www.praat.org.

[8] P. Boersma, "Accurate Short-Term Analysis of the Fundamental Frequency and the Harmonics-To-Noise Ratio of a Sampled Sound," *Proc. Inst. Phonetic Sci.*, vol. 17, pp. 97–110, 1993.

[9] Y. D. Heman-Ackah, D. D. Michael, and G. S. Goding, "The

relationship between cepstral peak prominence and selected parameters of dysphonia," *J. Voice*, vol. 16, no. 1, pp. 20–27, Mar. 2002, doi: 10.1016/S0892-1997(02)00067-X.

[10] R. Fraile and J. I. Godino-Llorente, "Cepstral peak prominence: A comprehensive analysis," *Biomed. Signal Process. Control*, vol. 14, pp. 42–54, 2014, doi: https://doi.org/10.1016/j.bspc.2014.07.001.

[11] M. Brockmann-Bauser, J. H. Van Stan, M. Carvalho Sampaio, J. E. Bohlender, R. E. Hillman, and D. D. Mehta, "Effects of Vocal Intensity and Fundamental Frequency on Cepstral Peak Prominence in Patients with Voice Disorders and Vocally Healthy Controls," *J. Voice*, 2019, doi: https://doi.org/10.1016/j.jvoice.2019.11.015.

[12] C. T. Ferrand, "Harmonics-to-noise ratio: An index of vocal aging," *J. Voice*, vol. 16, no. 4, pp. 480–487, Dec. 2002, doi: 10.1016/S0892-1997(02)00123-6.

[13] C. T. Ishi, K. I. Sakakibara, H. Ishiguro, and N. Hagita, "A method for automatic detection of vocal fry," *IEEE Trans. Audio, Speech Lang. Process.*, vol. 16, no. 1, pp. 47–56, 2008, doi: 10.1109/TASL.2007.910791.

[14] J. Kane, T. Drugman, and C. Gobl, "Improved automatic detection of creak," *Comput. Speech Lang.*, vol. 27, no. 4, pp. 1028–1047, Jun. 2013, doi: 10.1016/j.csl.2012.11.002.

[15] J. Hillenbrand, R. A. Cleveland, and R. L. Erickson, "Acoustic Correlates of Breathy Vocal Quality," *J. Speech, Lang. Hear. Res.*, vol. 37, no. 4, pp. 769–778, Aug. 1994, doi: 10.1044/jshr.3704.769.

[16] M. Brookes Voicebox: Speech processing toolbox for matlab, "Voicebox: Speech processing toolbox for matlab, Software available [Mar. 2011]." 1997, Accessed: May 05, 2020. [Online]. Available: http://www.ee.ic.ac.uk/hp/staff/dmb/voicebox/voicebox.html.

[17] R. L. Horwitz-Martin, "Acoustic features of impaired articulation due to amyotrophic lateral sclerosis," Harvard University/MIT, 2017.

[18] J. Sloboda, A. Lammert, J. Williamson, C. Smalt, D.D. Mehta, I. Curry, K. Heaton, J. Palmer, and T.F. Quatieri, "Vocal biomarkers for cognitive performance estimation in a working memory task," in *Proceedings of the Annual Conference of the International Speech Communication Association, INTERSPEECH*, 2018, pp. 1756–1760, doi: 10.21437/Interspeech.2018-2418.

[19] J. R. Williamson, T.F. Quatieri, A. Lammert, K. Mitchell, K. Finkelstein, N. Ekon, C. Dillon, R. Kenefick, and K. Heaton, "The effect of exposure to high altitude and heat on speech articulatory coordination," in *Proceedings of the Annual Conference of the International Speech Communication Association, INTERSPEECH*, 2018, pp. 297–301, doi: 10.21437/Interspeech.2018-2372.

[20] C. Espy-Wilson, A. Lammert, N. Seneviratne, and T. Quatieri, "Assessing neuromotor coordination in depression using inverted vocal tract variables," in *Proceedings of the Annual Conference of the International Speech Communication Association, INTERSPEECH*, 2019, pp. 1448–1452, doi: 10.21437/Interspeech.2019-1815.
